# Monitoring Steps and Heart Rate Using a Withings Smartwatch in Children and Adolescents With Cancer: Validation Study

**DOI:** 10.2196/77766

**Published:** 2026-06-10

**Authors:** Emma den Hartog, Marta Fiocco, Patrick van der Torre, Wim J E Tissing, Emma J Verwaaijen

**Affiliations:** 1 Princess Máxima Center for Pediatric Oncology, Heidelberglaan 25, Utrecht, 3584 CS, The Netherlands, 31 0889727272; 2 Mathematical Institute, Leiden University, Leiden, The Netherlands; 3 Department of Pediatric Oncology and Hematology, University of Groningen, Beatrix Children's Hospital, Groningen, The Netherlands

**Keywords:** validity, smartwatch, steps, heart rate, pediatric oncology

## Abstract

**Background:**

Consumer smartwatches offer potential for continuous monitoring of physical activity during childhood cancer treatment. However, their validity for measuring step count and heart rate in this population has not been established.

**Objective:**

This study examined the validity of a Withings smartwatch for monitoring step count and heart rate in children and adolescents undergoing cancer treatment.

**Methods:**

Children and adolescents aged 8 to 18 years under active cancer treatment wore a Withings smartwatch for 12 weeks. For validation purposes, children and adolescents simultaneously wore an ActiGraph wGT3X-BT accelerometer for 1 week under free-living conditions to assess step count agreement and were connected to a clinical heart rate monitor under resting conditions to assess heart rate agreement. Linear mixed models with participant ID as a random effect were used to estimate mean differences and derive 95% limits of agreement for repeated measurements.

**Results:**

A total of 14 children and adolescents participated in each of the validation analyses. The smartwatch recorded significantly fewer steps than the ActiGraph accelerometer for both steps per hour (mean difference 174 steps per hour; 95% limits of agreement=−283 to 633; *P*<.001) and steps per day (mean difference 3154 steps per day; 95% limits of agreement=−394 to 6702; *P*<.001), with increasing discrepancy at higher activity levels, suggesting proportional bias. No statistically significant difference in heart rate was observed between the smartwatch and the clinical heart rate monitor under resting conditions (mean difference −1.07 beats per minute; 95% limits of agreement=−17.55 to 15.41; *P*=.24).

**Conclusions:**

In children and adolescents undergoing cancer treatment, the Withings smartwatch showed minimal systematic bias for resting heart rate assessment, although individual variability was considerable. In contrast, step counts demonstrated systematic underestimation and wide limits of agreement compared with a research-grade accelerometer. The use of smartwatch-derived step counts for clinical benchmarking or goal setting in this population cannot currently be recommended.

## Introduction

Children undergoing treatment for cancer demonstrate substantially reduced levels of physical activity compared to healthy peers [1-3]. Disease- and treatment-related symptoms such as fatigue, musculoskeletal pain, and general malaise frequently limit mobility and contribute to muscle wasting, reduced functional capacity, and decreased participation in daily life [1-3]. Persistently low physical activity may contribute to a cycle of physical deconditioning, which can further impair functional independence, social participation, and quality of life [4,5]. In addition to physical consequences, reduced activity levels have been associated with adverse psychosocial outcomes, including reduced emotional well-being and diminished cognitive and social functioning [6-10].

Therefore, promoting and monitoring physical activity during treatment may not only address physical deconditioning but also support psychosocial functioning and quality of life. Given these multidimensional consequences, insight into physical activity behavior during treatment is clinically relevant for children with cancer, their families, and health care professionals. Monitoring physical activity may support early identification of functional decline, enable timely and individualized guidance, and inform shared decision-making regarding physical activity and rehabilitation strategies. However, such applications require accurate and feasible methods to assess physical activity under real-world conditions.

Objective measurement of physical activity in pediatric oncology relies primarily on research-grade accelerometers such as the ActiGraph, which are widely used as reference devices for quantifying physical activity, such as step count [11]. However, these devices do not provide information on physiological responses to activity, such as heart rate, and their use in routine clinical care is limited by practical considerations [12]. Data collection is usually restricted to predefined assessment periods; data are not directly accessible for children, parents, or health care professionals; and data extraction requires specialized software and expertise. Moreover, these devices may be experienced as inconvenient or uncomfortable by children. For these reasons, they are considered less suitable for continuous, long-term monitoring under free-living conditions [11].

Consumer-level wearable devices such as smartwatches offer the possibility to integrate measurement of physical activity (eg, step count) and physiological parameters such as heart rate within a single, low-burden device that can be worn continuously in daily life. This integration may provide a comprehensive picture of physical activity behavior during childhood cancer treatment while enabling real-time access to data for children, families, and health care professionals [11]. In this context, wearable devices have potential utility for monitoring activity patterns over time, supporting engagement in physical activity, and informing individualized guidance and rehabilitation strategies [13].

Several validation studies have evaluated consumer-grade wearable devices predominantly in adult populations, demonstrating promising accuracy for step count and heart rate under free-living conditions [14,15]. In pediatric populations, validation studies remain limited, and findings are heterogeneous [16]. Importantly, validation results obtained in adult or healthy pediatric populations cannot be readily generalized to children undergoing cancer treatment. In children with cancer, treatment-related symptoms, altered movement patterns, reduced gait speed, intermittent activity bouts, and physiological changes may influence sensor performance and algorithm accuracy. Therefore, device performance should be evaluated specifically within this population.

Among available consumer-grade devices, Withings smartwatches have frequently been used in research settings due to their suitability for research applications, including the availability of dedicated research infrastructure for data access and export and relatively stable software environments. Withings devices have been applied in pediatric studies to monitor physical activity behavior, primarily as tools to support physical activity interventions [17-19]. Although these devices have been validated in adult populations [17,20-25], validation has primarily focused on heart rate–related outcomes in pediatric populations, demonstrating acceptable agreement with reference methods [26]. Nonetheless, validation of Withings smartwatches in children undergoing cancer treatment in a wider context of physical activity has not yet been performed, and evidence regarding the validity of step count in any pediatric population remains limited.

Therefore, the aim of this study was to examine the validity of a Withings smartwatch for monitoring step count and heart rate in children and adolescents undergoing treatment for cancer.

## Methods

### Participants and Study Design

Participants were recruited within the framework of the Watch Us Move study, a prospective qualitative study examining the feasibility, barriers to, and facilitators of real-time and long-term physical activity tracking using a smartwatch in children and adolescents during treatment for cancer [18]. Eligible participants were aged 8 to 18 years; diagnosed with a malignancy; and receiving active treatment at the Princess Máxima Center for Pediatric Oncology, Utrecht, the Netherlands. Additional inclusion criteria were the ability to walk at least 50 meters without the use of a walking aid, adequate comprehension of the Dutch language, and access to a device capable of running the app. Exclusion criteria included children and adolescents who were scheduled to undergo stem cell transplantation during the study period or who had intellectual disabilities that precluded participation in long-term smartwatch use.

Eligible patients were identified during routine clinical visits between May 2023 and June 2024 and were approached by the research team in consultation with their treating pediatric oncologist or pediatric physiotherapist.

According to the design of the Watch Us Move study, children and adolescents wore a smartwatch for 12 weeks consecutively. For this substudy, children and adolescents were asked to wear the smartwatch and an ActiGraph (Ametris) accelerometer simultaneously for 1 week and keep a wear log and report nonwear periods throughout the week. Moreover, children and adolescents were asked to wear the smartwatch while connected to a heart rate monitor under resting conditions while being admitted to the day care treatment ward.

### Ethical Considerations

In accordance with Dutch legislation, for children aged 8 to 11 years, written informed consent was obtained from parents or legal guardians. For children and adolescents aged 12 to 15 years, written informed consent was obtained from both the participant and their parents or legal guardians. Adolescents aged 16 to 18 years provided written informed consent themselves. The study protocol was reviewed by the Medical Research Ethics Committee NedMec (reference number 23-003).

### Devices

#### Withings Smartwatch (Device Under Evaluation)

The Withings Steel HR and Pulse HR smartwatches were used as the device under evaluation in this study. These devices were selected for several methodological and practical reasons. First, Withings provides access to structured research data through a dedicated application programming interface, allowing for the secure retrieval of step count and heart rate data for analysis. In addition, the software environment of these devices is relatively stable, reducing the likelihood of major algorithm changes during the study period. Furthermore, Withings uses European data storage centers, which was considered important in light of the General Data Protection Regulation. Finally, the relatively small size, moderate cost, and design resembling a conventional wristwatch may enhance acceptability and feasibility for use in children and adolescents undergoing cancer treatment.

The Steel HR (model: HWA03b-36black-Inter or HWA03b-36white-Inter; 36.3 mm) is a hybrid smartwatch, and the Pulse HR (model: WAM03-Black Mirror-All-Inter; 18 × 44 mm) is a health and fitness tracker. Both devices use identical software for monitoring step count and heart rate.

Children and adolescents were allowed to choose the wrist on which they wore the Withings smartwatch to optimize comfort and adherence. When no preference was expressed, they were instructed to wear the device on the nondominant wrist in accordance with manufacturer recommendations. Wrist side was not systematically recorded.

Both devices were worn on the wrist and were capable of measuring step count, heart rate, distance covered, and estimated energy expenditure (calories). Data were synchronized with the Health Mate mobile app. Withings smartwatch data were manually retrieved from the Withings data server via the research data interface.

A day was considered valid when heart rate was recorded at least once per hour for a minimum of 6 hours between 6 AM and midnight. Nonwear time was defined as a period of at least 60 consecutive minutes without heart rate and step measurements. This definition was applied to ensure consistency with the nonwear definition used for the reference ActiGraph device (Troiano algorithm) [27]. For step count validation, only valid wear days were taken into account. For heart rate validation, the complete period during which children and adolescents were connected to the clinical heart rate monitor was considered.

#### ActiGraph (Reference Device for Step Count)

The ActiGraph wGT3X-BT accelerometer was used as the reference device for step count measurement under free-living conditions. The ActiGraph is widely used in pediatric research and, although it does not represent a criterion gold standard under free-living conditions, it is frequently applied as a research-grade comparator device in physical activity validation studies [28-30]. The ActiGraph was worn on the same wrist as the Withings smartwatch whenever possible. If this caused irritation or burden, it was worn on the opposite wrist. Wrist side (dominant vs nondominant) was not systematically recorded.

ActiGraph data were obtained using the ActiLife software (Ametris). A day was considered valid when steps were measured for at least 6 hours between 6 AM and midnight, with a minimum of 4 valid wear days per participant. Nonwear time was defined using the Troiano algorithm [27], in which a period of at least 60 consecutive minutes of zero activity counts was classified as nonwear, allowing for 1 to 2 minutes of activity counts between 0 and 100 [27]. Only valid wear days were included in step count analysis.

#### Philips IntelliVue X3 (Reference Device for Heart Rate)

The Philips IntelliVue X3 clinical monitor was used as the reference device for heart rate measurements. This hospital-grade monitoring system provides continuous electrocardiography-based heart rate measurements and is routinely used in clinical care. Therefore, it was considered an appropriate reference under resting clinical conditions. Children and adolescents were connected to the monitor under resting conditions while admitted to the day care treatment ward. Heart rate data were retrieved from the electronic patient records.

### Data Analysis

Descriptive statistics were used to summarize patient characteristics. Categorical variables were presented as numbers with percentages. Normally distributed continuous variables were reported as means with SDs, and nonnormally distributed data were presented as medians with IQRs or ranges.

Outcome measures included steps per hour (total number of steps), steps per day and heart rate per minute (beats per minute [bpm]). Measurements obtained using the smartwatch were compared with those from the reference devices (ActiGraph and heart rate monitor). Only days considered valid for both the smartwatch and ActiGraph were included in the step count analyses. All available heart rate measurements obtained during connection to the clinical monitor were included in the heart rate analysis.

Linear mixed-effects models were estimated to assess the mean difference between devices for each outcome measure. These models account for the presence of repeated measurements within participants. Participant ID was included as a random intercept. The fixed intercept of each model represents the estimated mean difference (bias) between devices.

For agreement analyses, 95% limits of agreement (LoA) were derived from the mixed-effects models. In accordance with recommendations for repeated-measure Bland-Altman analyses, the SD of the differences was calculated as the square root of the sum of the between- and within-subject variance components (ie, √[variance_participant + residual variance]) [31]. The LoA were then calculated as mean difference ± 1.96 × SD_total. Agreement was visually assessed using scatterplots and Bland-Altman plots. All analyses were performed in SPSS Statistics (version 29.0.1; IBM Corp) or R (version 4.4.1; R Foundation for Statistical Computing).

## Results

### Step Validation

**Figure 1.**
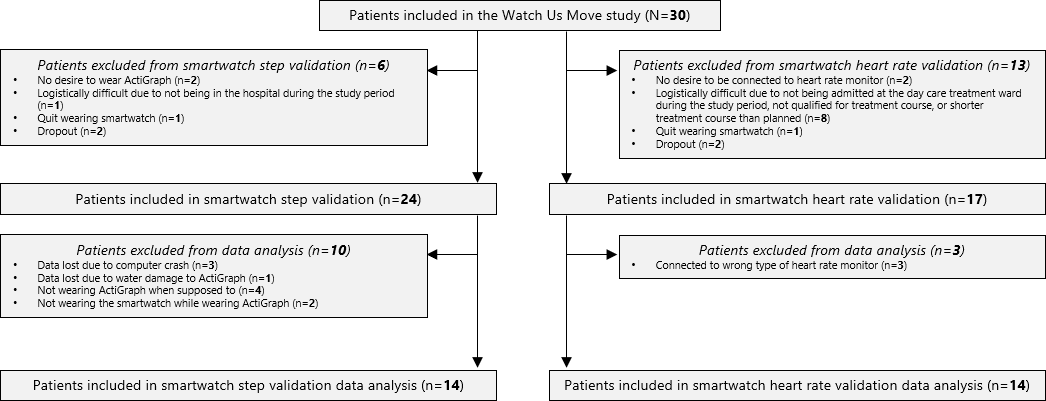
Flow diagram of the inclusion of patients.

During the study period, 14 children and adolescents wore the smartwatch simultaneously while wearing an ActiGraph in free-living conditions for 1 week (Figure 1). Of these 14 children and adolescents, 8 (57.1%) were female, and they had a median age of 13.5 (IQR 10.5-15.3) years and weight-to-height *Z* score of 0 (IQR −1.5 to 1.6; Table 1).

**Table 1.** Patient characteristics and free-living step count data during the step count validation study (n=14).

Characteristic	Values
Sex, n (%)	
Female	8 (57.1)
Male	6 (42.9)
Age (y), median (IQR)	13.5 (10.5 to 15.3)
Time since diagnosis (d), median (IQR)	108.5 (63.8 to 201.0)
Diagnosis, n (%)	
Acute lymphoblastic leukemia	6 (42.9)
Lymphoblastic lymphoma	3 (21.4)
Hodgkin lymphoma	1 (7.1)
Medulloblastoma	2 (14.3)
Osteosarcoma	1 (7.1)
Ewing sarcoma	1 (7.1)
Weight-to-height *Z* score, median (IQR)	0 (–1.5 to 1.6)
Free-living step count during the validation week, median (range)	
Steps per h measured via the ActiGraph	188 (0 to 4725)
Steps per h measured via the smartwatch	33 (0 to 4799)
Steps per d measured via the ActiGraph	5697 (1598 to 17,436)
Steps per d measured via the smartwatch	2213 (12 to 12,425)

During the 1-week study period in free-living conditions, the median number of steps per hour was 188 (range 0-4725) as measured using the ActiGraph and 33 (range 0-4799) as measured using the smartwatch. The median steps per day measured using the ActiGraph and the smartwatch were 5697 (range 1598-17,436) and 2213 (range 12-12,425), respectively.

**Figure 2.**
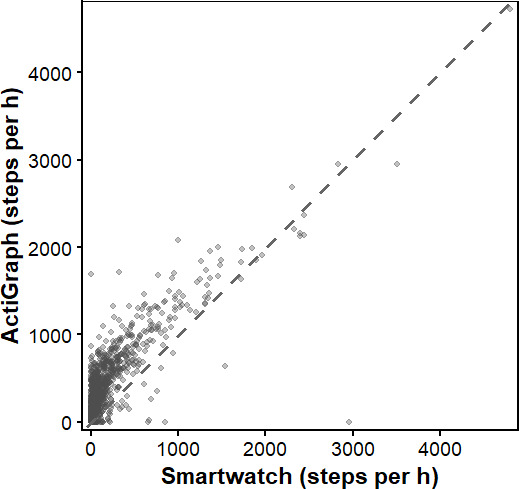
Scatterplot comparing smartwatch and ActiGraph steps per hour. The dashed line represents the line of equality (*y*=*x*).

Results from the linear mixed model revealed a significant difference in steps per hour between the ActiGraph and the smartwatch (*P*<.001). The mean difference (ActiGraph – smartwatch) was 174 steps per hour. The 95% LoA ranged from −283 to 633 steps per hour. Figures2  3 visualize the repeated measurements and agreement for steps per hour.

**Figure 3.**
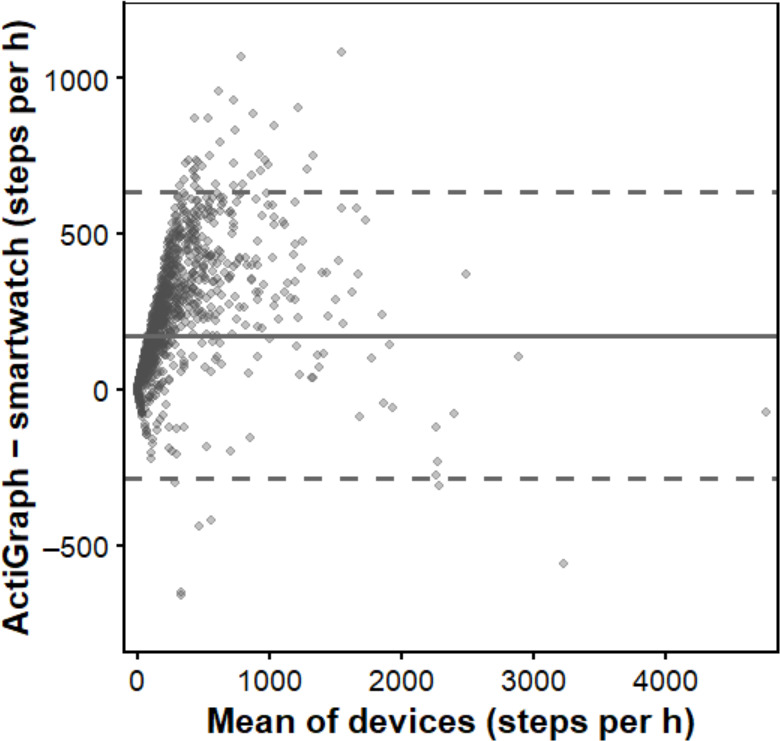
Bland-Altman plot for steps per hour comparing the smartwatch and ActiGraph. The solid line indicates the mean difference; the dashed lines represent the 95% limits of agreement.

**Figure 4.**
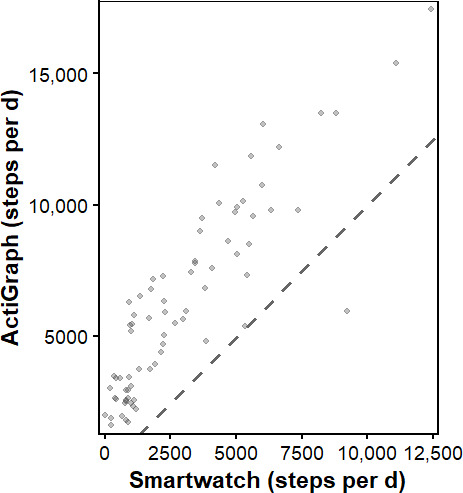
Scatterplot comparing smartwatch and ActiGraph steps per day. The dashed line represents the line of equality (*y*=*x*).

Similarly, for steps per day, a significant difference between devices was observed (*P*<.001). The mean difference was 3154 steps per day, with 95% LoA ranging from −394 to 6702 steps per day. Figures4  5 illustrate the agreement for steps per day. With increasing average hourly as well as daily step counts, the difference between devices increased, suggesting proportional bias.

**Figure 5.**
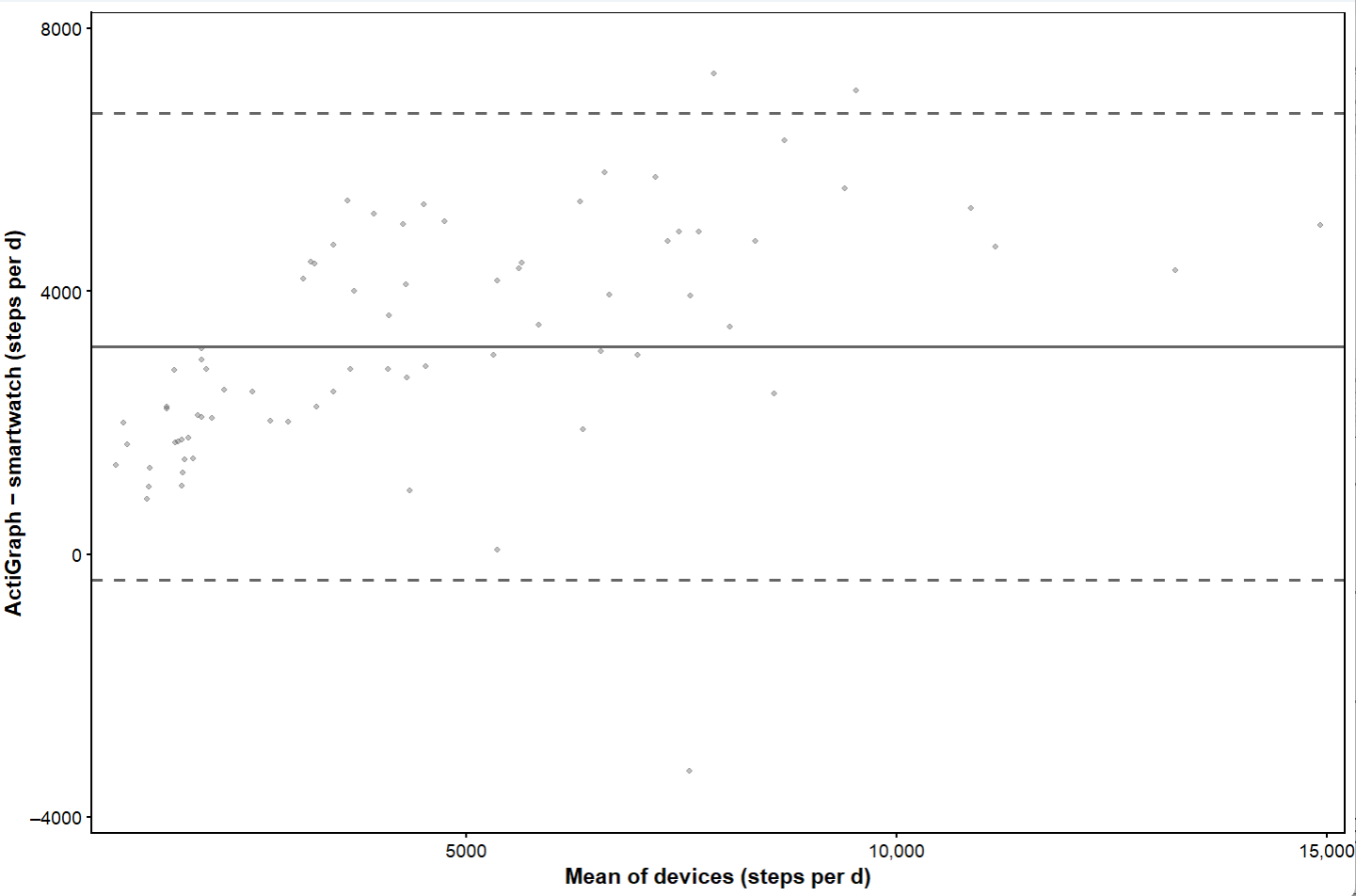
Bland-Altman plot for steps per day comparing the smartwatch and ActiGraph. The solid line indicates the mean difference; the dashed lines represent the 95% limits of agreement.

### Heart Rate Validation

During the study period, 14 children and adolescents wore the smartwatch while being connected to a clinical heart rate monitor under resting conditions (Figure 1). In total, 42.9% (n=6) of them were female, with a median age of 12.5 (IQR 9.0-16.0) years and a weight-to-height *Z* score of 0.1 (IQR −1.2 to 1.1; Table 2).

**Table 2.** Patient characteristics and resting heart rate data during the heart rate validation study (n=14).

Characteristic	Values
Sex, n (%)	
Female	6 (42.9)
Male	8 (57.1)
Age (y), median (IQR)	12.5 (9.0 to 16.0)
Time since diagnosis (d), median (IQR)	95.0 (74.8 to 140.8)
Diagnosis, n (%)	
Acute lymphoblastic leukemia	9 (64.3)
Lymphoblastic lymphoma	3 (21.4)
Osteosarcoma	1 (7.1)
Ewing sarcoma	1 (7.1)
Weight-to-height *Z* score, median (IQR)	0.1 (−1.2 to 1.1)
Resting heart rate measurements	
Duration of heart rate measurement period, range	6 min to 3 h, 43 min
Heart rate measured via the heart rate monitor (bpm^a^), median (range)	83 (47 to 115)
Heart rate measured via the smartwatch (bpm), median (range)	85 (46 to 113)

a bpm: beats per minute.

During the resting measurement period at the day care treatment ward (range 6 minutes-3 hours 43 minutes), the median heart rate measured using the clinical monitor and the smartwatch was 83 (range 47-115) bpm and 85 (range 46-113) bpm, respectively.

**Figure 6.**
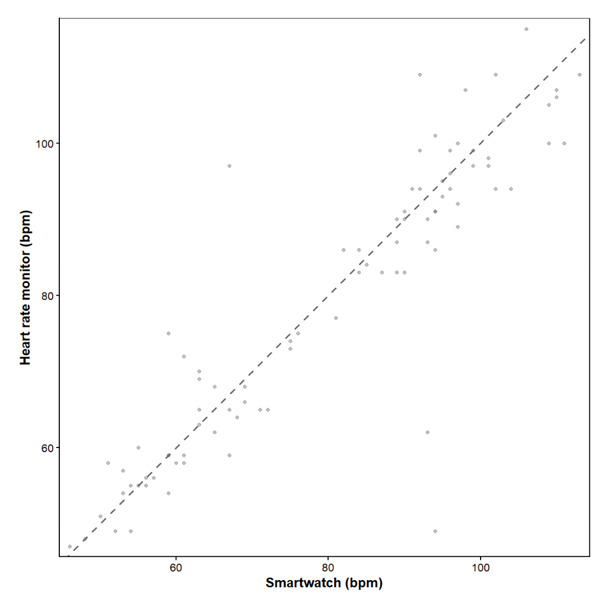
Scatterplot comparing smartwatch and clinical heart rate monitor beats per minute (bpm). The dashed line represents the line of equality (*y*=*x*).

The linear mixed model analysis revealed no statistically significant difference between devices (*P*=.24). The mean difference (monitor – smartwatch) was −1.07 bpm. The 95% LoA ranged from −17.55 to 15.41 bpm. Figures6  7 illustrate the agreement between devices for heart rate per minute.

**Figure 7.**
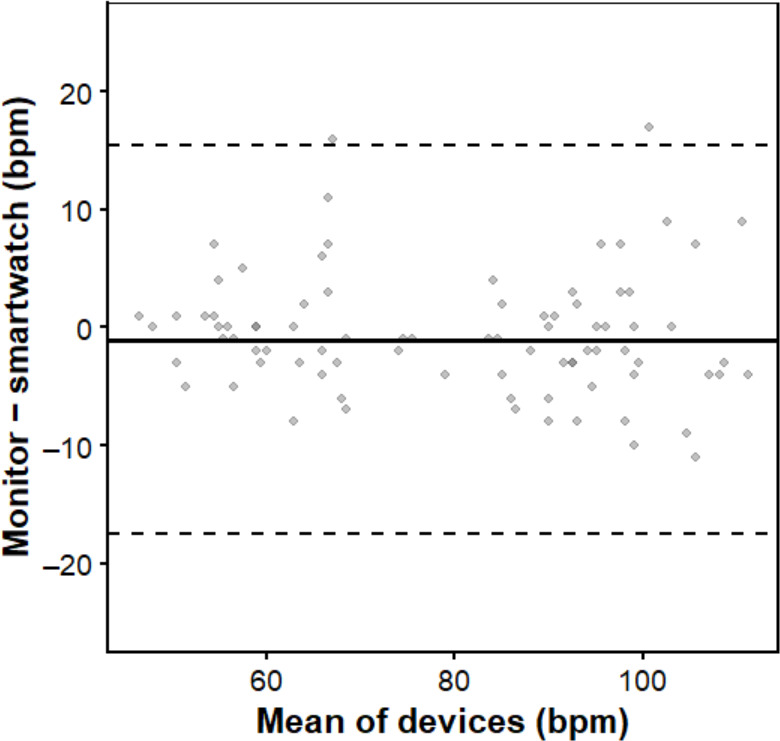
Bland-Altman plot for heart rate beats per minute (bpm) comparing the smartwatch and clinical heart rate monitor. The solid line indicates the mean difference; the dashed lines represent the 95% limits of agreement.

## Discussion

### Principal Findings

In this validation study, the Withings smartwatch systematically recorded fewer steps than the wrist-worn ActiGraph under free-living conditions in children and adolescents undergoing cancer treatment. In contrast, resting heart rate measurements showed minimal systematic bias, although individual variability remained considerable.

The smartwatch consistently recorded fewer steps than the ActiGraph, and the magnitude of this discrepancy increased with higher activity levels, indicating proportional bias. This suggests that the difference between devices was not constant across the activity spectrum but became more pronounced at higher step counts. As a result, more active children may be disproportionately affected by underestimation, and step counts derived from the smartwatch may not accurately reflect relative differences between children. In addition, the wide LoA observed in this study indicate substantial variability at the individual level. Together, these findings reinforce that smartwatch-derived step counts should not be considered interchangeable with those obtained from research-grade accelerometers, particularly when absolute step counts are used for clinical benchmarking or comparison across studies. A similar pattern has been described in older adults at risk of functional decline, where a Withings smartwatch also underestimated step count compared with a wrist-worn ActiGraph under free-living conditions [32].

Several factors may contribute to these observed differences. Wrist-worn accelerometers are sensitive to nonambulatory arm movements, which may lead to overestimation of step count when worn on the wrist. Conversely, consumer-grade smartwatches may apply proprietary filtering algorithms to minimize false-positive step detection, potentially resulting in underestimation of low-intensity or irregular movement patterns [32,33]. In addition, differences in step detection thresholds and algorithm design between devices likely contribute to systematic discrepancies. Importantly, as no criterion gold standard for step count under free-living conditions was available, the observed discrepancy should be interpreted as a difference between device algorithms and placements rather than definitive measurement error of one device alone.

In the broader literature, consumer activity trackers have often shown good associations with research-grade accelerometers for daily step counts in pediatric populations [34-36]. However, studies have also reported systematic differences in absolute step counts, particularly at higher activity intensities [34,36,37]. For example, Fitbit devices have been found to provide comparable step estimates at low intensities but to overestimate steps during moderate to vigorous physical activity [34]. Children with cancer may exhibit altered gait patterns, intermittent low-intensity activity bouts, and treatment-related movement alterations, conditions under which wrist-based step detection algorithms may be particularly challenged. Our findings indicate that device-related discrepancies were also present in children and adolescents undergoing cancer treatment, highlighting that associations do not necessarily imply agreement in absolute step values.

In contrast to step count, heart rate measurements obtained under resting clinical conditions via the smartwatch showed minimal systematic bias compared with the reference monitor. The mean difference was small (approximately −1 bpm), indicating that group average resting heart rate values were similar between devices. However, LoA ranging from approximately −18 to 15 bpm indicate variability at the individual level, which should be considered when interpreting individual heart rate measurements. These findings are consistent with those of previous validation studies in adult populations, including patients scheduled for elective surgery and healthy adults, in which Withings smartwatches demonstrated good agreement with chest strap sensors or clinical monitors under low-intensity or resting conditions [24,38]. However, it should be noted that heart rate was assessed only under relatively stable, low-intensity conditions and within a limited heart rate range in our study. The validity of smartwatch-derived heart rate measurements during higher-intensity activities or in free-living conditions remains uncertain.

From a clinical perspective, wearable monitors may have several potential applications in pediatric oncology. Continuous monitoring can provide insights into activity quantity (eg, daily step count) as well as patterns over time (eg, diurnal variation, day-to-day fluctuations, and recovery after hospitalization), which may be difficult to capture through periodic assessments. However, the observed systematic and proportional differences in step count between devices are particularly relevant in pediatric oncology, where subtle changes in activity may reflect recovery, treatment tolerance, or emerging complications. If higher activity levels are systematically underestimated, improvements during rehabilitation or recovery phases may be less visible when relying solely on smartwatch-derived step counts. In this context, inaccurate step feedback may influence motivation and self-perception of performance. Underestimation at higher activity levels could be particularly discouraging for children who actively engage in rehabilitation or physical activity goals [18]. Therefore, interpretation of wearable-derived step data should focus primarily on intraindividual trends rather than absolute comparisons with normative thresholds or research-grade accelerometers. In this context, within-person trends over time using the same device may be more informative than absolute comparisons with predefined step thresholds or research-grade accelerometers. Such longitudinal information may support early recognition of functional decline, facilitate individualized counseling by physiotherapists, and help evaluate adherence to physical activity recommendations or rehabilitation plans. In addition, feedback from wearable devices may support motivation and engagement in an active lifestyle for some children, although this may also be confronting for others and should be used cautiously and in a supportive manner [18].

This validation study has several limitations that need to be taken into account when interpreting the results. First, no criterion gold standard for step count under free-living conditions was available. The ActiGraph was used as a widely accepted research-grade comparator. However, wrist-worn accelerometers have been shown to overestimate step count, particularly under conditions of low-intensity or irregular movement [39]. In addition, differences between devices may reflect variation in sensitivity to wrist movement, step detection thresholds, and algorithm design. Therefore, the observed discrepancy between devices may represent characteristics of both measurement systems rather than true measurement error. Second, the exact threshold values and proprietary algorithms used by both devices to define a step are not publicly available. Differences in filtering strategies may partly explain both the systematic underestimation and the proportional bias observed in the smartwatch. Third, nonwear time for the ActiGraph was determined using the Troiano algorithm [27]. In children undergoing cancer treatment, prolonged periods of very low–intensity activity are common [1-3], which makes it challenging to distinguish inactivity from nonwear using standard algorithms. This may have influenced step count comparisons, particularly at lower activity levels, where misclassification of wear time may occur. Fourth, wrist side (dominant vs nondominant) was not systematically recorded. Children and adolescents were allowed to choose the wrist to optimize comfort and adherence, which reflects real-world implementation but may have introduced additional variability in step estimates. Although dominant wrist placement has been shown to influence step counts [40], previously reported interwrist differences are generally smaller than the discrepancy observed in our study. Nevertheless, we cannot fully exclude some contribution of limb dominance. Fifth, heart rate was validated only under resting clinical conditions within a limited heart rate range and, therefore, cannot be generalized to higher-intensity activities or free-living conditions where motion artifacts and greater variability may affect smartwatch performance. In addition, disease- and treatment-related factors (eg, treatment phase, hospitalization, fatigue, neuropathy, reduced gait speed, and intermittent activity bouts) may alter movement patterns and physiological responses. Our sample size did not allow for stratified analyses by clinical characteristics. Hence, device agreement may differ across subgroups. Finally, the exploratory nature of this study and the relatively small sample size limit the precision of the estimated LoA and reduce generalizability.

Future research should aim to further elucidate the mechanisms underlying the observed disagreement in step count between devices. Inclusion of additional criterion measures, such as direct observation or controlled laboratory-based step assessments, may help determine whether discrepancies arise primarily from overestimation by wrist-worn accelerometers, underestimation by smartwatch algorithms, or a combination of both. While waist-worn accelerometers may provide complementary insights into the influence of upper-limb movement on step detection, their feasibility for long-term monitoring in pediatric oncology is limited, and therefore, they are primarily relevant as methodological reference tools rather than implementation devices. Given the observed proportional bias, future studies should also examine whether calibration approaches or device-specific correction models could improve agreement across different activity intensities. In addition, validation of smartwatch-derived heart rate measurements under free-living conditions and across a broader physiological range is warranted. Larger studies across different treatment phases are needed to explore whether disease- and treatment-related characteristics influence device performance. Furthermore, evaluating test-retest reliability and responsiveness over time will be essential to determine whether smartwatch-derived measures are suitable for longitudinal monitoring of physical activity trajectories during and after treatment.

### Conclusions

In this exploratory validation study, the Withings smartwatch showed minimal systematic bias for resting heart rate under controlled clinical conditions, although individual variability was considerable. In contrast, smartwatch-derived step counts were consistently lower than those obtained using a research-grade accelerometer and showed increasing disagreement at higher activity levels. Given the observed systematic and proportional discrepancies, use of smartwatch-derived step counts for clinical benchmarking or goal setting in children undergoing cancer treatment cannot currently be recommended.

## Abbreviations

bpm: beats per minute
LoA: limits of agreement
